# Application of four-dimension criteria to assess rigour of qualitative research in emergency medicine

**DOI:** 10.1186/s12913-018-2915-2

**Published:** 2018-02-17

**Authors:** Roberto Forero, Shizar Nahidi, Josephine De Costa, Mohammed Mohsin, Gerry Fitzgerald, Nick Gibson, Sally McCarthy, Patrick Aboagye-Sarfo

**Affiliations:** 1 The Simpson Centre for Health Services Research, South Western Sydney Clinical School and the Ingham Institute for Applied Research, Liverpool Hospital, UNSW, Liverpool, NSW 1871 Australia; 2 Psychiatry Research and Teaching Unit, Liverpool Hospital, NSW Health, Sydney, Australia; 3 School of Psychiatry, Faculty of Medicine, University of New South Wales, Sydney, Australia; 4 School - Public Health and Social Work, Queensland University of Technology (QUT), Brisbane, Qld Australia; 5 Australasian College for Emergency Medicine (ACEM), Melbourne, VIC Australia; 6 School of Nursing and Midwifery, Edith Cowan University (ECU), Perth, WA Australia; 7 Emergency Care Institute (ECI), NSW Agency for Clinical Innovation (ACI), Sydney, Australia; 8 Clinical Support Directorate, System Policy & Planning Division, Department of Health WA, Perth, WA Australia

**Keywords:** Emergency department, Four-hour rule, Australia, Policy assessment, Qualitative methods, Interviews, Research design

## Abstract

**Background:**

The main objective of this methodological manuscript was to illustrate the role of using qualitative research in emergency settings. We outline rigorous criteria applied to a qualitative study assessing perceptions and experiences of staff working in Australian emergency departments.

**Methods:**

We used an integrated mixed-methodology framework to identify different perspectives and experiences of emergency department staff during the implementation of a time target government policy. The qualitative study comprised interviews from 119 participants across 16 hospitals. The interviews were conducted in 2015–2016 and the data were managed using NVivo version 11. We conducted the analysis in three stages, namely: conceptual framework, comparison and contrast and hypothesis development. We concluded with the implementation of the four-dimension criteria (credibility, dependability, confirmability and transferability) to assess the robustness of the study,

**Results:**

We adapted four-dimension criteria to assess the rigour of a large-scale qualitative research in the emergency department context. The criteria comprised strategies such as building the research team; preparing data collection guidelines; defining and obtaining adequate participation; reaching data saturation and ensuring high levels of consistency and inter-coder agreement.

**Conclusion:**

Based on the findings, the proposed framework satisfied the four-dimension criteria and generated potential qualitative research applications to emergency medicine research. We have added a methodological contribution to the ongoing debate about rigour in qualitative research which we hope will guide future studies in this topic in emergency care research. It also provided recommendations for conducting future mixed-methods studies. Future papers on this series will use the results from qualitative data and the empirical findings from longitudinal data linkage to further identify factors associated with ED performance; they will be reported separately.

**Electronic supplementary material:**

The online version of this article (10.1186/s12913-018-2915-2) contains supplementary material, which is available to authorized users.

## Background

Qualitative research methods have been used in emergency settings in a variety of ways to address important problems that cannot be explored in another way, such as attitudes, preferences and reasons for presenting to the emergency department (ED) versus other type of clinical services (i.e., general practice) [1–4].

The methodological contribution of this research is part of the ongoing debate of scientific rigour in emergency care, such as the importance of qualitative research in evidence-based medicine, its contribution to tool development and policy evaluation [2, 3, 5–7]. For instance, the Four-Hour Rule and the National Emergency Access Target (4HR/NEAT) was an important policy implemented in Australia to reduce EDs crowding and boarding (access block) [8–13]. This policy generated the right conditions for using mixed methods to investigate the impact of 4HR/NEAT policy implementation on people attending, working or managing this type of problems in emergency departments [2, 3, 5–7, 14–17].

The rationale of our study was to address the perennial question of how to assess and establish methodological robustness in these types of studies. For that reason, we conducted this mixed method study to explore the impact of the 4HR/NEAT in 16 metropolitan hospitals in four Australian states and territories, namely: Western Australia (WA), Queensland (QLD), New South Wales (NSW), and the Australian Capital Territory (ACT) [18, 19].

The main objectives of the qualitative component was to understand the personal, professional and organisational perspectives reported by ED staff during the implementation of 4HR/NEAT, and to explore their perceptions and experiences associated with the implementation of the policy in their local environment.

This is part of an Australian National Health and Medical Research Council (NH&MRC) Partnership project to assess the impact of the 4HR/NEAT on Australian EDs. It is intended to complement the quantitative streams of a large data-linkage/dynamic modelling study using a mixed-methods approach to understand the impact of the implementation of the four-hour rule policy.

## Methods

### Methodological rigour

This section describes the qualitative methods to assess the rigour of the qualitative study. Researchers conducting quantitative studies use conventional terms such as internal validity, reliability, objectivity and external validity [17]. In establishing trustworthiness, Lincoln and Guba created stringent criteria in qualitative research, known as credibility, dependability, confirmability and transferability [17–20]. This is referred in this article as “the Four-Dimensions Criteria” (FDC). Other studies have used different variations of these categories to stablish rigour [18, 19]. In our case, we adapted the criteria point by point by selecting those strategies that applied to our study systematically. Table 1 illustrates which strategies were adapted in our study.

**Table 1:** Key FDC strategies adapted from Lincoln and Guba [23]

Rigour Criteria	Purpose	Original Strategies	Strategies applied in our study to achieve rigour
Credibility	To establish confidence that the results (from the perspective of the participants) are true, credible and believable.	• Prolonged and varied engagement with each setting	• Interviewers spent an average of 6–8 weeks per site to engage with ED and participants.
		• Interviewing process and techniques	• Interview protocol tested at two induction meetings and using 1–2 pilot interviews.
		• Establishing investigators’ authority	• We ensured the investigators had the required knowledge and research skills to perform their roles.
		• Collection of referential adequacy materials	• We asked interviewers to send all the field notes to the research office for analysis and storage.
		• Peer debriefing	• We had regular debriefing sessions with key members of Project Management Committee/Australasian College of Emergency Medicine.
Dependability	To ensure the findings of this qualitative inquiry are repeatable if the inquiry occurred within the same cohort of participants, coders and context.	• Rich description of the study methods	• We prepared detailed drafts of the study protocol throughout the study.
		• Establishing an audit trail	• We developed a detailed track record of the data collection process.
		• Stepwise replication of the data	• We measured coding accuracy and inter-coders’ reliability of the research team.
Confirmability	To extend the confidence that the results would be confirmed or corroborated by other researchers.	• Reflexivity	• We implemented reflexive journals and weekly investigators meetings.
		• Triangulation	• We applied several triangulation techniques (methodological, data source, investigators and theoretical).
Transferability	To extend the degree to which the results can be generalized or transferred to other contexts or settings.	• Purposeful sampling to form a nominated sample	• We used a combination of three purposive sampling techniques.
		• Data saturation	• We quantified operational and theoretical data saturation.

### Study procedure

We carefully planned and conducted a series of semi-structured interviews based on the four-dimension criteria (credibility, dependability, confirmability and transferability) to assess and ensure the robustness of the study. These criteria have been used in other contexts of qualitative health research; but this is the first time it has been used in the emergency setting [20–26].

### Sampling and recruitment

We employed a combination of stratified purposive sampling (quota sampling), criterion-based and maximum variation sampling strategies to recruit potential participants [27, 28]. The hospitals selected for the main longitudinal quantitative data linkage study, were also purposively selected in this qualitative component.

We targeted potential individuals from four groups, namely: ED Directors, ED physicians, ED nurses, and data/admin staff. The investigators identified local site coordinators who arranged the recruitment in each of the participating 16 hospitals (6 in NSW, 4 in QLD, 4 in WA and 2 in the ACT) and facilitated on-site access to the research team. These coordinators provided a list of potential participants for each professional group. By using this list, participants within each group were selected through purposive sampling technique. We initially planned to recruit at least one ED director, two ED physicians, two ED nurses and one data/admin staff per hospital. Invitation emails were circulated by the site coordinators to all potential participants who were asked to contact the main investigators if they required more information.

We also employed criterion-based purposive sampling to ensure that those with experience relating to 4HR/NEAT were eligible. For ethical on-site restrictions, the primary condition of the inclusion criteria was that eligible participants needed to be working in the ED during the period that the 4HR/NEAT policy was implemented. Those who were not working in that ED during the implementation period were not eligible to participate, even if they had previous working experience in other EDs.

We used maximum variation sampling to ensure that the sample reflects a diverse group in terms of skill level, professional experience and policy implementation [28]. We included study participants irrespective of whether their role/position was changed (for example, if they received a promotion during their term of service in ED).

In summary, over a period of 7 months (August 2015 to March 2016), we identified all the potential participants (124) and conducted 119 interviews (5 were unable to participate due to workload availability). The overall sample comprised a cohort of people working in different roles across 16 hospitals. Table 2 presents the demographic and professional characteristics of the participants.

**Table 2:** Demographic and professional characteristics of the staff participated in the study

Characteristics	Number of participants (*n* = 119)	% of total participants
Gender		
Male	57	48
Female	62	52
Role in the ED		
ED Directors (ED Dir/Deputy Dir/Acting Dir)	21	18
ED Physicians (Staff Spec, Registrars)	43	36
ED Nurses (NUMs, CNCs, Nurse Coordinator)	44	37
Data or Administrator (data managers, admin)	11	9
Time of service in ED (years)		
Mean	13.5	
Range	3–33	
Median	12	
State/territory of service		
NSW/ACT	52	44
QLD	37	31
WA	30	25

*Dir* represents ‘Director’, *NUM* Nursing unit manager, *CNC* Clinical nurse consultant

### Data collection

We employed a semi-structured interview technique. Six experienced investigators (3 in NSW, 1 in ACT, 1 in QLD and 1 in WA) conducted the interviews (117 face-to-face on site and 2 by telephone). We used an integrated interview protocol which consisted of a demographically-oriented question and six open-ended questions about different aspects of the 4HR/NEAT policy (see Additional file 1: Appendix 1).

With the participant’s permission, interviews were audio-recorded. All the hospitals provided a quiet interview room that ensured privacy and confidentiality for participants and investigators.

All the interviews were transcribed verbatim by a professional transcriber with reference to a standardised transcription protocol [29]. The data analysis team followed a stepwise process for data cleaning, and de-identification. Transcripts were imported to qualitative data analysis software NVivo version 11 for management and coding [30].

### Data analysis

**Fig. 1:**
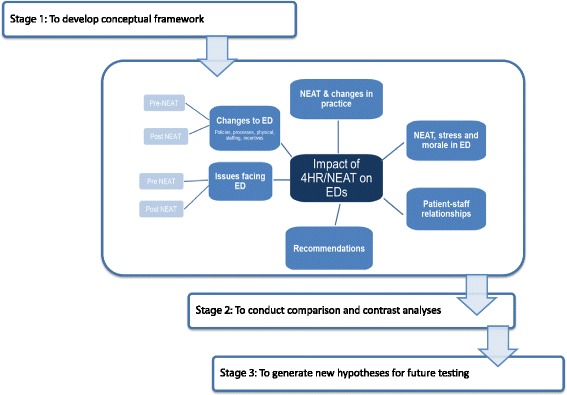
Conceptual framework with the three stages of analysis used for the analysis of the qualitative data

The analyses were carried out in three stages. In the first stage, we identified key concepts using content analysis and a mind-mapping process from the research protocol and developed a conceptual framework to organise the data [31]. The analysis team reviewed and coded a selected number of transcripts, then juxtaposed the codes against the domains incorporated in the interview protocol as indicated in the three stages of analysis with the conceptual framework (Fig. 1).

In this stage, two cycles of coding were conducted: in the first one, all the transcripts were revised and initially coded, key concepts were identified throughout the full data set. The second cycle comprised an in-depth exploration and creation of additional categories to generate the codebook (see Additional file 2: Appendix 2). This codebook was a summary document encompassing all the concepts identified as primary and subsequent levels. It presented hierarchical categorisation of key concepts developed from the domains indicated in Fig. 1.

A summarised list of key concepts and their definitions are presented in Table 3. We show the total number of interviews for each of the key concepts, and the number of times (i.e., total citations) a concept appeared in the whole dataset.

**Table 3:** Summary of key concepts, their definition, total number of citations and total number of interviews

	Key concepts	Definition of key concepts based on participants’ information	Interviews	Total citations
1	Relationships	Discussion of interactions between ED staff and staff from elsewhere in the hospital, and how these relationships affected and were affected by 4HR/NEAT.	119	5308
2	Characteristics of care in EDs pre- and post-4HR/NEAT	Participants discussed about the characteristics of care in ED before after 4HR/NEAT, and how 4HR/NEAT have changed these characteristics along its implementation.	119	1920
3	Staffing and 4HR/NEAT	Participants’ references to the impact and influence of 4HR/NEAT on staffing. These include creating new roles, changing/shuffling the responsibilities, issues around staff shortage and supply after 4HR/NEAT, etc.	113	1457
4	Recommendations	Participants expressed and explained a number of recommendations based on their experience with 4HR/NEAT implementation. These were suggested to be taken on board if other hospitals/organisations intended to adopt and implement time targets.	113	1070
5	Access block	Interactions and relationships reported in relation to 4HR/NEAT performance and access block as a principal factor associated with ED overcrowding.	109	910
6	4HR/NEAT introduction & management	Concepts and descriptions relevant to the introduction of 4HR/NEAT into hospitals and how this was managed.	106	880
7	External factors	Participants described a number of factors on which ED has had no control. It included all the factors imposed to ED from the department of health or the hospital executives (e.g., budget cut and changes to the hospital services that influenced ED operation).	95	868
8	Changes to ED related to 4HR/NEAT	All the references and explanations relating to the changes that were brought in as a result of 4HR/NEAT implementation.	115	565
9	4HR/NEAT performance	References how participants thought their hospital performed in terms of meeting the 4HR/NEAT target.	103	429
10	Medical education and training	Participants’ explanation of how and to what extent 4HR/NEAT have had an influence on medical education and training of ED staff.	46	206

Citations refer to the number of times a coded term was counted in NVivo

The second stage of analysis compared and contrasted the experiences, perspectives and actions of participants by role and location. The third and final stage of analysis aimed to generate theory-driven hypotheses and provided an in-depth understanding of the impact of the policy. At this stage, the research team explored different theoretical perspectives such as the carousel model and models of care approach [16, 32–34]. We also used iterative sampling to reach saturation and interpret the findings.

### Ethics approval and consent to participate

Ethics approval was obtained for all participating hospitals and the qualitative methods are based on the original research protocol approved by the funding organisations [18].

## Results

This section described the FDC and provided a detailed description of the strategies used in the analysis. It was adapted from the FDC methodology described by Lincoln and Guba [23–26] as the framework to ensure a high level of rigour in qualitative research. In Table 1, we have provided examples of how the process was implemented for each criterion and techniques to ensure compliance with the purpose of FDC.

### Credibility

#### Prolonged and varied engagement with each setting

All the investigators had the opportunity to have a continued engagement with each ED during the data collection process. They received a supporting material package, comprising background information about the project; consent forms and the interview protocol (see Additional file 1: Appendix 1). They were introduced to each setting by the local coordinator and had the chance to meet the ED directors and potential participants, They also identified local issues and salient characteristics of each site, and had time to get acquainted with the study’s participants. This process allowed the investigators to check their personal perspectives and predispositions, and enhance their familiarity with the study setting. This strategy also allowed participants to become familiar with the project and the research team.

#### Interviewing process and techniques

In order to increase credibility of the data collected and of the subsequent results, we took a further step of calibrating the level of awareness and knowledge of the research protocol. The research team conducted training sessions, teleconferences, induction meetings and pilot interviews with the local coordinators. Each of the interviewers conducted one or two pilot interviews to refine the overall process using the interview protocol, time-management and the overall running of the interviews.

The semi-structured interview procedure also allowed focus and flexibility during the interviews. The interview protocol (Additional file 1: Appendix 1) included several prompts that allowed the expansion of answers and the opportunity for requesting more information, if required.

#### Establishing investigators’ authority

In relation to credibility, Miles and Huberman [35] expanded the concept to the trustworthiness of investigators’ authority as ‘human instruments’ and recommended the research team should present the following characteristics:

- *Familiarity with phenomenon and research context*: In our study, the research team had several years’ experience in the development and implementation of 4HR/NEAT in Australian EDs and extensive ED-based research experience and track records conducting this type of work.
- *Investigative skills:* Investigators who were involved in data collections had three or more years’ experience in conducting qualitative data collection, specifically individual interview techniques.
- *Theoretical knowledge and skills in conceptualising large datasets:* Investigators had post-graduate experience in qualitative data analysis and using NVivo software to manage and qualitative research skills to code and interpret large amounts of qualitative data.
- *Ability to take a multidisciplinary approach:* The multidisciplinary background of the team in public health, nursing, emergency medicine, health promotion, social sciences, epidemiology and health services research, enabled us to explore different theoretical perspectives and using an eclectic approach to interpret the findings.

These characteristics ensured that the data collection and content were consistent across states and participating hospitals.

#### Collection of referential adequacy materials

In accordance with Guba’s recommendation to collect any additional relevant resources, investigators maintained a separate set of materials from on-site data collection which included documents and field notes that provided additional information in relation to the context of the study, its findings and interpretation of results. These materials were collected and used during the different levels of data analysis and kept for future reference and secure storage of confidential material [26].

#### Peer debriefing

We conducted several sessions of peer debriefing with some of the Project Management Committee (PMC) members. They were asked at different stages throughout the analysis to reflect and cast their views on the conceptual analysis framework, the key concepts identified during the first level of analysis and eventually the whole set of findings (see Fig. 1). We also have reported and discussed preliminary methods and general findings at several scientific meetings of the Australasian College for Emergency Medicine.

### Dependability

#### Rich description of the study protocol

This study was developed from the early stages through a systematic search of the existing literature about the four-hour rule and time-target care delivery in ED. Detailed draft of the study protocol was delivered in consultation with the PMC. After incorporating all the comments, a final draft was generated for the purpose of obtaining the required ethics approvals for each ED setting in different states and territories.

To maintain consistency, we documented all the changes and revisions to the research protocol, and kept a trackable record of when and how changes were implemented.

#### Establishing an audit trail

Steps were taken to keep a track record of the data collection process [24]: we have had sustained communication within the research team to ensure the interviewers were abiding by an agreed-upon protocol to recruit participants. As indicated before, we provided the investigators with a supporting material package. We also instructed the interviewers on how to securely transfer the data to the transcriber. The data-analysis team systematically reviewed the transcripts against the audio files for accuracy and clarifications provided by the transcriber.

All the steps in coding the data and identification of key concepts were agreed upon by the research team. The progress of the data analysis was monitored on a weekly basis. Any modifications of the coding system were discussed and verified by the team to ensure correct and consistent interpretation throughout the analysis.

The codebook (see Additional file 2: Appendix 2) was revised and updated during the cycles of coding. Utilisation of the mind-mapping process described above helped to verify consistency and allowed to determine how precise the participants’ original information was preserved in the coding [31].

As required by relevant Australian legislation [36], we maintained complete records of the correspondence and minutes of meetings, as well as all qualitative data files in NVivo and Excel on the administrative organisation’s secure drive. Back-up files were kept in a secure external storage device, for future access if required.

#### Stepwise replication—measuring the inter-coders’ agreement

To assess the interpretative rigour of the analysis, we applied inter-coder agreement to control the coding accuracy and monitor inter-coder reliability among the research team throughout the analysis stage [37]. This step was crucially important in the study given the changes of staff that our team experienced during the analysis stage. At the initial stages of coding, we tested the inter-coder agreement using the following protocol:

- Step 1 – Two data analysts and principal investigator coded six interviews, separately.
- Step 2 – The team discussed the interpretation of the emerging key concepts, and resolved any coding discrepancies.
- Step 3 – The initial codebook was composed and used for developing the respective conceptual framework.
- Step 4 – The inter-coder agreement was calculated and found a weighted Kappa coefficient of 0.765 which indicates a very good agreement (76.5%) of the data.

With the addition of a new analyst to the team, we applied another round of inter-coder agreement assessment. We followed the same steps to ensure the inter-coder reliability along the trajectory of data analysis, except for step 3—a priori codebook was used as a benchmark to compare and contrast the codes developed by the new analyst. The calculated Kappa coefficient 0.822 indicates a very good agreement of the data (See Table 4).

**Table 4:** Inter-coder analysis using Cohen’s Kappa coefficients

	Key concepts	1st team of coders (initial stage)		2nd team of coders (later stage)	
		Kappa (unweighted)	Kappa (weighted)	Kappa (unweighted)	Kappa (weighted)
1	Relationships	0.772	**0.750**	0.806	**0.805**
2	Characteristics of care in EDs pre- and post-4HR/NEAT	0.825	**0.837**	0.720	**0.726**
3	Staffing and 4HR/NEAT	0.978	**0.974**	0.794	**0.808**
4	Recommendations	0.772	**0.691**	0.875	**0.864**
5	Access block	0.909	**0.914**	0.948	**0.949**
6	4HR/NEAT introduction and management	0.786	**0.724**	0.810	**0.820**
7	External factors	0.710	**0.707**	0.874	**0.871**
8	Changes to ED related to 4HR/NEAT	0.792	**0.775**	0.815	**0.816**
9	4HR/NEAT performance	0.912	**0.905**	0.700	**0.702**
10	Medical education and training	0.789	**0.632**	0.855	**0.865**
Overall Kappa		–	**0.765**	–	**0.822**

### Confirmability

#### Reflexivity

The analysis was conducted by the research team who brought different perspectives to the data interpretation. To appreciate the collective interpretation of the findings, each investigator used a separate reflexive journal to record the issues about sensitive topics or any potential ethical issues that might have affected the data analysis. These were discussed in the weekly  meetings.

After completion of the data collection, reflection and feedback from all the investigators conducting the interviews were sought in both written and verbal format.

#### Triangulation

To assess the confirmability and credibility of the findings, the following four triangulation processes were considered: methodological, data source, investigators and theoretical triangulation.

Methodological triangulation is in the process of being implemented using the mixed methods approach with linked data from our 16 hospitals.

Data source triangulation was achieved by using several groups of ED staff working in different states/territories and performing different roles. This triangulation offered a broad source of data that contributed to gain a holistic understanding of the impact of 4HR/NEAT on EDs across Australia. We expect to use data triangulation with linked-data in future secondary analysis.

Investigators triangulation was obtained by consensus decision making though collaboration, discussion and participation of the team holding different perspectives. We also used the investigators’ field notes, memos and reflexive journals as a form of triangulation to validate the data collected. This approach enabled us to balance out the potential bias of individual investigators and enabling the research team to reach a satisfactory consensus level.

Theoretical triangulation was achieved by using and exploring different theoretical perspectives such as the carousel model and models of care approach [16, 32–34]. that could be applied in the context of the study to generate hypotheses and theory driven codes [16, 32, 38].

### Transferability

#### Purposive sampling to form a nominated sample

As outlined in the methods section, we used a combination of three purposive sampling techniques to make sure that the selected participants were representative of the variety of views of ED staff across settings. This representativeness was critical for conducting comparative analysis across different groups.

#### Data saturation

We employed two methods to ensure data saturation was reached, namely: operational and theoretical. The operational method was used to quantify the number of new codes per interview over time. It indicates that the majority of codes were identified in the first interviews, followed by a decreasing frequency of codes identified from other interviews.

**Fig. 2:**
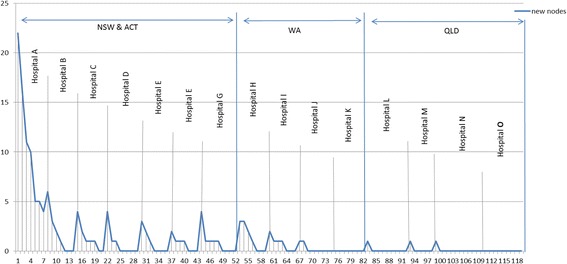
Data saturation gain per interview added based on the chronological order of data collection in the hospitals. Y axis = number of new codes, X axis = number of interviews over time

Theoretical saturation and iterative sampling were achieved through regular meetings where progress of coding and identification of variations in each of the key concepts were reported and discussed. We also used iterative sampling to reach saturation and interpret the findings. We continued this iterative process until no new codes emerged from the dataset and all the variations of an observed phenomenon were identified [39] (Fig. 2).

## Discussion

Scientific rigour in qualitative research assessing trustworthiness is not new. Qualitative researchers have used rigour criteria widely [40–42]. The novelty of the method described in this article rests on the systematic application of these criteria in a large-scale qualitative study in the context of emergency medicine.

According to the FDC, similar findings should be obtained if the process is repeated with the same cohort of participants in the same settings and organisational context. By employing the FDC and the proposed strategies, we could enhance the dependability of the findings. As indicated in the literature, qualitative research has many times been questioned in history for its validity and credibility [3, 20, 43, 44].

Nevertheless, if the work is done properly, based on the suggested tools and techniques, any qualitative work can become a solid piece of evidence. This study suggests that emergency medicine researchers can improve their qualitative research if conducted according to the suggested criteria. The triangulation and reflexivity strategies helped us to minimise the investigators’ bias, and affirm that the findings were objective and accurately reflect the participants’ perspectives and experiences. Abiding by a consistent method of data collection (e.g., interview protocol) and conducting the analysis with a team of investigators, helped us minimise the risk of interpretation bias.

Employing several purposive sampling techniques enabled us to have a diverse range of opinions and experiences which at the same time enhanced the credibility of the findings. We expect that the outcomes of this study will show a high degree of applicability, because any resultant hypotheses may be transferable across similar settings in emergency care. The systematic quantification of data saturation at this scale of qualitative data has not been demonstrated in the emergency medicine literature before.

As indicated, the objective of this study was to contribute to the ongoing debate about rigour in qualitative research by using our mixed methods study as an example. In relation to innovative application of mixed-methods, the findings from this qualitative component can be used to explain specific findings from the quantitative component of the study. For example, different trends of 4HR/NEAT performance can be explained by variations in staff relationships across states (see key concept 1, Table 3). In addition, some experiences from doctors and nurses may explain variability of performance indicators across participating hospitals. The robustness of the qualitative data will allow us to generate hypotheses that in turn can be tested in future research.

Careful planning is essential in any type of research project which includes the importance of allocating sufficient resources both human and financial. It is also required to organise precise arrangements for building the research team; preparing data collection guidelines; defining and obtaining adequate participation. This may allow other researchers in emergency care to replicate the use of the FDC in the future.

This study has several limitations. Some limitations of the qualitative component include recall bias or lack of reliable information collected about interventions conducted in the past (before the implementation of the policy). As Weber and colleagues [45] point out, conducting interviews with clinicians at a single point in time may be affected by recall bias. Moreover, ED staff may have left the organisation or have progressed in their careers (from junior to senior clinical roles, i.e. junior nursing staff or junior medical officers, registrars, etc.), so obtaining information about pre/during/post-4HR/NEAT was a difficult undertaking. Although the use of criterion-based and maximum-variation sampling techniques minimised this effect, we could not guarantee that the sampling techniques could have reached out all those who might be eligible to participate.

In terms of recruitment, we could not select potential participants who were not working in that particular ED during the implementation, even if they had previous working experience in other hospital EDs. This is a limitation because people who participated in previous hospitals during the intervention could not provide valuable input to the overall project.

In addition, one would claim that the findings could have been ‘ED-biased’ due to the fact that we did not interview the staff or administrators outside the ED. Unfortunately, interviews outside the ED were beyond the resources and scope of the project.

With respect to the rigour criteria, we could not carry out a systematic member checking as we did not have the required resources for such an expensive follow-up. Nevertheless, we have taken extensive measures to ensure confirmation of the integrity of the data.

## Conclusions

The FDC presented in this manuscript provides an important and systematic approach to achieve trustworthy qualitative findings. As indicated before, qualitative research credentials have been questioned. However, if the work is done properly based on the suggested tools and techniques described in this manuscript, any work can become a very notable piece of evidence. This study concludes that the FDC is effective; any investigator in emergency medicine research can improve their qualitative research if conducted accordingly.

Important indicators such as saturation levels and inter-coder reliability should be considered in all types of qualitative projects. One important aspect is that by using FDC we can demonstrate that qualitative research is not less rigorous than quantitative methods.

We also conclude that the FDC is a valid framework to be used in qualitative research in the emergency medicine context. We recommend that future research in emergency care should consider the FDC to achieve trustworthy qualitative findings. We can conclude that our method confirms the credibility (validity) and dependability (reliability) of the analysis which are a true reflection of the perspectives reported by the group of participants across different states/territories.

We can also conclude that our method confirms the objectivity of the analyses and reduces the risk for interpretation bias. We encourage adherence to practical frameworks and strategies like those presented in this manuscript.

Finally, we have highlighted the importance of allocating sufficient resources. This is essential if other researchers in emergency care would like to replicate the use of the FDC in the future.

Following papers in this series will use the empirical findings from longitudinal data linkage analyses and the results from the qualitative study to further identify factors associated with ED performance before and after the implementation of the 4HR/NEAT.

## Additional files

Additional file 1: **Appendix 1.** Interview form. Text. (PDF 445 kb)

Additional file 2: **Appendix 2.** Codebook NVIVO. Text code. (PDF 335 kb)

Additional file 3: **Appendix 3.** Acknowledgements. Text. (PDF 104 kb)

## Abbreviations

4HR/NEAT: Four Hour Rule/National Emergency Access Target
ACT: Australian Capital Territory
ED/EDs: Emergency department(s)
FDC: Four-Dimensions Criteria
HREC: Health Research Ethics Committee
NSW: New South Wales
PMC: Project Management Committee
QLD: Queensland
WA: Western Australia

## References

[1] Rees N, Rapport F, Snooks H (2015). Perceptions of paramedics and emergency staff about the care they provide to people who self-harm: constructivist metasynthesis of the qualitative literature. J Psychosom Res.

[2] Jessup M, Crilly J, Boyle J, Wallis M, Lind J, Green D, Fitzgerald G (2016). Users’ experiences of an emergency department patient admission predictive tool: a qualitative evaluation. Health Inform J.

[3] Choo EK, Garro AC, Ranney ML, Meisel ZF, Morrow Guthrie K (2015). Qualitative research in emergency care part I: research principles and common applications. Acad Emerg Med.

[4] Hjortdahl M, Halvorsen P, Risor MB (2016). Rural GPs’ attitudes toward participating in emergency medicine: a qualitative study. Scand J Prim Health Care.

[5] Samuels-Kalow ME, Rhodes KV, Henien M, Hardy E, Moore T, Wong F, Camargo CA, Rizzo CT, Mollen C (2017). Development of a patient-centered outcome measure for emergency department asthma patients. Acad Emerg Med.

[6] Manning SN (2016). A multiple case study of patient journeys in Wales from A&E to a hospital ward or home. Br J Community Nurs.

[7] Ranney ML, Meisel ZF, Choo EK, Garro AC, Sasson C, Morrow Guthrie K (2015). Interview-based qualitative research in emergency care part II: data collection, analysis and results reporting. Acad Emerg Med.

[8] Forero R, Hillman KM, McCarthy S, Fatovich DM, Joseph AP, Richardson DB (2010). Access block and ED overcrowding. Emerg Med Australas.

[9] Fatovich DM (2003). Access block: problems and progress. Med J Aust.

[10] Richardson DB (2006). Increase in patient mortality at 10 days associated with emergency department overcrowding. Med J Aust.

[11] Sprivulis PC, Da Silva J-A, Jacobs IG, Frazer ARL, Jelinek GA (2006). The association between hospital overcrowding and mortality among patients admitted via Western Australian emergency departments.[Erratum appears in Med J Aust. 2006 Jun 19;184(12):616]. Med J Aust.

[12] Richardson DB, Mountain D (2009). Myths versus facts in emergency department overcrowding and hospital access block. Med J Aust.

[13] Geelhoed GC, de Klerk NH (2012). Emergency department overcrowding, mortality and the 4-hour rule in Western Australia. Med J Aust.

[14] Nugus P, Forero R (2011). Understanding interdepartmental and organizational work in the emergency department: an ethnographic approach. Int Emerg Nurs.

[15] Nugus P, Holdgate A, Fry M, Forero R, McCarthy S, Braithwaite J (2011). Work pressure and patient flow management in the emergency department: findings from an ethnographic study. Acad Emerg Med.

[16] Nugus P, Forero R, McCarthy S, McDonnell G, Travaglia J, Hilman K, Braithwaite J (2014). The emergency department “carousel”: an ethnographically-derived model of the dynamics of patient flow. Inte Emerg Nurs.

[17] Jones P, Chalmers L, Wells S, Ameratunga S, Carswell P, Ashton T, Curtis E, Reid P, Stewart J, Harper A (2012). Implementing performance improvement in New Zealand emergency departments: the six hour time target policy national research project protocol. BMC Health Serv Res.

[18] Forero R, Hillman K, McDonnell G, Fatovich D, McCarthy S, Mountain D, Sprivulis P, Celenza A, Tridgell P, Mohsin M (2013). Validation and impact of the four hour rule/NEAT in the emergency department: a large data linkage study (Grant # APP1029492). Vol. $687,000.

[19] Forero R, Hillman K, McDonnell G, Tridgell P, Gibson N, Sprivulis P, Mohsin M, Green S, Fatovich D, McCarthy S (2013). Study Protocol to assess the implementation of the Four-Hour National Emergency Access Target (NEAT) in Australian Emergency Departments.

[20] Morse JM (2015). Critical analysis of strategies for determining rigor in qualitative inquiry. Qual Health Res.

[21] Schou L, Hostrup H, Lyngso EE, Larsen S, Poulsen I (2012). Validation of a new assessment tool for qualitative research articles. J Adv Nurs.

[22] Tuckett AG (2005). Part II. Rigour in qualitative research: complexities and solutions. Nurse Res.

[23] Lincoln YS, Guba EG (1986). But is it rigorous? Trustworthiness and authenticity in naturalistic evaluation. N Dir Eval.

[24] Lincoln YS, Guba EG. Naturalistic inquiry, 1st edn. Newbury Park: Sage Publications Inc; 1985.

[25] Guba EG, Lincoln YS (1982). Epistemological and methodological bases of naturalistic inquiry. ECTJ.

[26] Guba EG (1981). Criteria for assessing the trustworthiness of naturalistic inquiries. ECTJ.

[27] Palinkas LA, Horwitz SM, Green CA, Wisdom JP, Duan N, Hoagwood K (2015). Purposeful sampling for qualitative data collection and analysis in mixed method implementation research. Adm Policy Ment Health Ment Health Serv Res.

[28] Patton MQ. Qualitative research and evaluation methods. 3rd edn. Thousand Oaks: Sage Publishing; 2002.

[29] McLellan E, MacQueen KM, Neidig JL (2003). Beyond the qualitative interview: data preparation and transcription. Field Methods.

[30] QSR International Pty Ltd (2015). NVivo 11 for windows.

[31] Whiting M, Sines D (2012). Mind maps: establishing ‘trustworthiness’ in qualitative research. Nurse Res.

[32] Forero R, Nugus P, McDonnell G, McCarthy S, Deng M, Raia F, Vaccarella M (2012). Iron meets clay in sculpturing emergency medicine: a multidisciplinary sense making approach. Relational concepts in medicine (eBook).

[33] FitzGerald G, Toloo GS, Romeo M (2014). Emergency healthcare of the future. Emerg Med Australas.

[34] NSW Ministry of Health, Health NDo (2012). Emergency department models of care.

[35] Miles MB, Huberman AM. Qualitative data analysis: an expanded sourcebook: Sage; 1994.

[36] Council NHaMR. In: Council NHaMR, editor. Australian code for the responsible conduct of research: Australian Government; 2007.

[37] Kuckartz U. Qualitative text analysis: a guide to methods, practice and using software: Sage; 2014.

[38] Ulrich W, Reynolds M (2010). Critical systems heuristics. Systems approaches to managing change: a practical guide.

[39] Bowen GA (2008). Naturalistic inquiry and the saturation concept: a research note. Qual Res.

[40] Krefting L (1991). Rigor in qualitative research - the assessment of trustworthiness. Am J Occup Ther.

[41] Hamberg K, Johansson E, Lindgren G, Westman G (1994). Scientific rigour in qualitative research - examples from a study of womens health in family-practice. Fam Pract.

[42] Tobin GA, Begley CM (2004). Methodological rigour within a qualitative framework. J Adv Nurs.

[43] Kitto SC, Chesters J, Grbich C (2008). Quality in qualitative research. Med J Aust.

[44] Tuckett AG, Stewart DE (2003). Collecting qualitative data: part I: journal as a method: experience, rationale and limitations. Contemp Nurse.

[45] Weber EJ, Mason S, Carter A, Hew RL (2011). Emptying the corridors of shame: organizational lessons from England’s 4-hour emergency throughput target. Ann Emerg Med.

