# Supplementary material for: Application of four-dimension criteria to assess rigour of qualitative research in emergency medicine
Source: BMC Health Serv Res. 2018 Feb 17;18:120. doi: 10.1186/s12913-018-2915-2 (PMC5816375; doi:10.1186/s12913-018-2915-2)
Supplement: Supplementary file 1 — Appendix 1. Interview form. Text. (PDF 445 kb) [file 12913_2018_2915_MOESM1_ESM.pdf]

4HR/NEAT Project

Interview Schedule:

Qualitative interview – 60 minutes

The aim of this research is to understand how the four hour rule/NEAT has had an impact on participating Emergency Departments and hospitals across WA, NSW, QLD and ACT.

1. **Background information**:
   a. Can you tell me your position here (and how long you have worked in this position at this hospital)?
   b. Have you held any other positions here?
   c. Overall, how long you have been working at [hospital] ED?

2. What do ***you*** think about the target?
   - From your personal perspective
   - From your professional perspective (e.g. how has it enabled or held back your performance of your role in the ED?)
   - From your organisation's perspective (e.g. how well has your ED performed in meeting the target?)

3. Based on ***your*** experience prior to the target's introduction, what difference, if any, has the target made to:
   a. ***Your*** practice?
   b. *For managerial staff only*: The ***hospital*** you manage?

4. What were the most important issues facing your ED that hindered performance:
   a. ***Two to five years prior*** to the implementation of the 4hr NEAT?
   b. ***During*** implementation of the 4hr NEAT?
   c. *Now*?

Some possible examples:

- Staffing issues
- Access Block
- Patient Care
- Compliance with the NEAT
- Completeness of care

5. What ***changes*** were planned ***before*** the target was implemented (**DATE**), and what has been done ***since*** its implementation? (Please give us **dates** for these changes where you can.)

Some possible examples:

- Physical changes to ED
- Staff role/rostering changes
- Process changes (triage, admission/discharge process, radiology)
- *Managerial staff*: ***policy*** level changes?
- *Managerial staff*: ***Financial incentives***/funding changes?

6. What has been the effect of the target on ***ED staff*** with regard to stress/morale, and how has this had an impact on ***patients*** and ***staff relationships with patients***?

Some possible examples:

- Has workload pressure increased/decreased/remained the same as a result of NEAT?
- Has NEAT affected the flow of power through clinical and professional relationships of its staff?
- Has moving patients through the system at a faster rate affected staff interactions/relationships with patients?
- How do you think patients feel about the NEAT?

7. What advice would ***you*** give other organisations trying to make these kinds of process changes?

8. *For managerial staff only*: We would like to have an overall picture of your hospital's ED, could you fill out the following table:
   *(This table will be filled out by **one** member of the **managerial or data manager** staff **per hospital**)*

Provide answers for the most recent calendar year (2014):

Total attendances<br>• *By ATS categories*	
Total ambulance attendances	
Total paediatric attendances (Age < or equal to 15) as per ACEM survey	
Total Admissions<br>• *Of these, ED short stay admissions*	
Total treatment cubicles/locations<br>• *Of these– ambulant , non-ambulant cubicles/locations*	
ED short stay beds	
Total hospital overnight beds (including overnight ED short stay beds)	
Typical clinical ED FTE per day<br>• *Of these Medical, Nursing, Allied Health*	
Typical ATS Category waiting time compliance for each category	
Typical 4 hour rule compliance for ED short stay admissions	
Typical 4 hour rule compliance for non-ED (i.e. inpatient) admissions	
Typical ambulance diversion performance *(circle)*	Not allowed in this State<br><br>Less than an hour a day<br><br>More than an hour a day
Typical ambulance ramping issues (i.e. ambulance unloading delayed by more than 15 minutes due to a lack of space in the ED to unload patient) *(circle)*	Less than weekly<br><br>At least once a week<br><br>Most days of the week
