# Supplementary material for: Application of four-dimension criteria to assess rigour of qualitative research in emergency medicine
Source: BMC Health Serv Res. 2018 Feb 17;18:120. doi: 10.1186/s12913-018-2915-2 (PMC5816375; doi:10.1186/s12913-018-2915-2)
Supplement: Supplementary file 2 — Appendix 2. Codebook NVIVO. Text code. (PDF 335 kb) [file 12913_2018_2915_MOESM2_ESM.pdf]

Codebook for Qualitative Component –Four Hour Rule Project

(Short version: including key concepts and first level of codes)

Key concepts and codes	Interviews	References	Definitions based on participants' information
• **Relationships**	**119**	**5308**	**Discussion of a wide range of interactions between ED staff and staff from elsewhere in the hospital, and how these relationships affected and were affected by NEAT.**
ED and Stress and Morale	116	1476	All the references to and explanations of how the level of stress and morale has been influenced by the implementation of NEAT
NEAT and Patients	115	1539	Effect of NEAT implementation on patients.
NEAT and the 'Whole of Hospital' Approach	103	606	Participant references to "whole of hospital" approaches to achieving NEAT.
ED-Other Wards Relationships	100	508	Participants' references to the issues, problems and opportunities in the context of ED staff's relationships with those in the inpatient teams or other wards.
Relationships within ED staff	93	566	Participant refers to relationships (whether positive or negative) between ED staff, including doctors/nurses/data administrators, etc.
ED-Executive Relationships	92	531	Participant discusses the relationship between ED staff members and members of the hospital's administration/executive.
ED and Bullying	18	84	Participants suggested that treating staff in an overbearing or intimidating manner has been detected between some ED staff (e.g., nurses and junior doctors) as well as between ED and other wards/specialties.

**Key concepts and codes** (continued)	**Interviews**	**References**	**Definitions based on participants' information**
• **Characteristics of care in EDs pre- and post-4HR/NEAT**	**119**	**1920**	**Participants discussed about the characteristics of care in ED before after NEAT, and how NEAT has changes these characteristics along its implementation.**
Impacts of NEAT on ED Care and Processes	119	1688	Participant discusses issues around the care that patients receive in ED after the introduction of NEAT.
General	85	232	Participant generally discusses issues around how ED cares for patients.
• **Changes to ED related to 4HR/NEAT**	**115**	**565**	**All the references and explanations relating to the changes that were brought in as a result of NEAT implementation.**
NEAT Implementation - Models of Care	103	278	For example: the Front-loaded assessment, team-based care, Rapid Assessment Team, etc. which were applied during the implementation of NEAT in order to meeting the target.
NEAT implementation - Policy changes	63	133	Participants referred to and explained a number of policies that were adopted by hospitals or EDs as a mean to improve their performances.
Gaming	29	44	Manipulation of NEAT data to meet target
NEAT implementation – Process changes in inpatient teams	26	47	Any process changes that was adopted and practised by the inpatient teams to help meet the target.
NEAT implementation – IT system	18	25	Implementation and application of a variety of IT systems during NEAT implementation.
NEAT Implementation - Enhancing the capacity and layout of ED	13	15	Reported changes to the layout of the ED in order to enhance the capacity of the department.
NEAT implementations - Others interventions and changes	5	5	A general discussion from participants in which a variety of NEAT implementations and strategies are listed (e.g., rostering changes)

Key concepts and codes (continued)	Interviews	References	Definitions based on participants' information
• **Staffing and 4HR/NEAT**	**113**	**1457**	**Participant's references to the impact and influence of NEAT on staffing. These include creating new roles, changing/shuffling the responsibilities, issues around staff shortage and supply after NEAT, etc.**
Staff shortages and supply	96	602	Not having enough staff on the floor to meet the day-to-day demands of the ED
NEAT-related staff roles	89	722	Changes to staffing roles related to the NEAT such as "Navigator Nurse" role.
Rostering changes	39	108	Any changes to rostering shifts in favour of meeting the target
Allied health's input in ED process	13	16	The influence of allied health services on ED decision making and how it impacts (delays) the admission or discharge of patients from ED, and contributes to the overall patient flow in ED.
• **Recommendations**	**113**	**1070**	**Recommendations and suggestions based on participants' experience with NEAT implementation. These include all the take-home messages for other hospitals/organisations intending to adopt and implement time targets.**
NEAT and Issues with Data & Measurement	77	322	Participants' recommendations and suggestions in terms of the problems and downsides of setting up measurement tools to measure NEAT performance, and how these should be tailored or amended.
Resourcing of EDs and NEAT	58	322	There needs to be more funding to support any changes to the hospital/ED
Recommendations on staff and executives buy-in	55	131	Specific recommendations with regards to the necessity and importance of having the executive buy-in , along with the buy-in from all levels of staff in the Ed and hospital.
Specific Recommendations to Change NEAT Goals	52	246	To make NEAT practicable, the target should be broken down into small palatable bits.
General Recommendations & Opinions	35	46	Participant's general recommendations and suggestions with regards to how to improve performance and meet NEAT.

Key concepts and codes (continued)	Interviews	References	Definitions based on participants' information
• **Access Block**	**109**	**910**	**Interactions and relationships reported in relation to NEAT performance and access block as a principal factor associated with ED overcrowding.**
Access block along the trajectory of NEAT	82	243	Participants perception of changes to access block before, during and after NEAT implementation.
Access block generally	58	139	Participants' general discussion about access block and some of its key associates.
Consequences of access block	57	145	Reported and speculated consequences of having access block in the Ed and hospital.
Contributing factors to access block	40	71	Participants' explanation of the factors contributing to either amelioration or improvement of access block in the hospitals.
Access block dictated changes to the ED processes	16	18	Some participants believe that it was access block that precipitated or urged changes to the ED.
• **4HR/NEAT Introduction and Management**	**106**	**880**	**References to the introduction of NEAT into hospitals and how this was managed by directors or management.**
Managing Staff Understanding of NEAT	65	303	Participant discusses issues around staff and their understanding of NEAT requirements.
NEAT Financial Incentives	57	189	Participants' views on financial incentives offered by the Government for meeting the target.
Change Management	52	370	Explanations and difficulties of change management in EDs during NEAT implementation.
NEAT as a KPI	14	18	The Overall and admission NEAT were viewed as a KPI that measures the patient flow through ED, and indicates the organisation's stress, but does not measure the quality of care.

**Key concepts and codes** (continued)	**Interviews**	**References**	**Definitions based on participants' information**
• **4HR/NEAT Performance**	**103**	**429**	**Participants' perceptions about their hospital performance in terms of meeting NEAT targets, and what factors have been mentioned as contributing to their hospital/ED performance against target either positive or negative.**
NEAT performance improved but the target is not met	38	78	Participants noted that NEAT performance improved but the target is not met due to a number of reasons (see child nodes)
NEAT performance improved initially but plateaued or failed	29	78	Participants noted that NEAT performance improved initially but it failed or plateaued due to a number of reasons (see child nodes)
Unsatisfactory NEAT performance	27	62	Participants perceived their NEAT performance being unsatisfactory. A number of reasons were reported in association with the unsatisfactory NEAT performances of their ED (see child nodes).
Satisfactory NEAT performance	23	41	Participants perceived their NEAT performance being satisfactory. A number of reasons were also disclosed as contribution factors to the perceived satisfactory NEAT performances of their ED (see child nodes).
Issues with NEAT performance measurement	2	4	Reported issues regarding the measurement of ED's performance against NEAT.
• **External Factors**	**95**	**868**	**Participants described a number of factors on which ED has had no control. It includes all the factors imposed to ED from the department of health or the hospital executives (e.g., budget cut and changes to the hospital services that influenced ED's operation).**
Capacity and Physical Size of the ED	80	418	Pre-and post-NEAT issues relating to the design and capacity of the ED to properly accommodate and serve patients.
Increasing Patient Presentations	68	288	The increasing number of presentations caused or mediated the access block.

**Key concepts and codes** (continued)	**Interviews**	**References**	**Definitions based on participants' information**
Budget Cuts	25	78	References to post-NEAT budget cuts that caused, e.g. a decrease in the number of ED staff (common in WA interviews).
ED Workload Has Increased	23	60	ED staff overworked and stressed both pre and post NEAT due to a range of factors such as increasing number of presentations, documentation , etc.
Phasing-out of Hospital Services	4	23	Explanation of how reducing the hospital services and closing some of the wards can influence the ED performance with regards to the NEAT target.
• **Medical education and training**	**46**	**206**	**Participants' reports on how medical education and training of ED staff was influenced by the implementation of NEAT.**
NEAT Negatively Impacted Medical Education & Training	27	83	Participant's general opinions and comments about how medical education and training in the ED discipline has been negatively influenced as a result of NEAT implementation.
NEAT Positively Impacted Medical Education & Training	13	36	Participant's general opinions and comments about how medical education and training in the ED discipline has been positively influenced as a result of NEAT implementation.
General Discussions Around Medical Education & Training in ED	12	15	Participant's general opinions and comments about how medical education and training in the ED discipline has been changed as a result of NEAT implementation.
