# Supplementary material for: Application of four-dimension criteria to assess rigour of qualitative research in emergency medicine
Source: BMC Health Serv Res. 2018 Feb 17;18:120. doi: 10.1186/s12913-018-2915-2 (PMC5816375; doi:10.1186/s12913-018-2915-2)
Supplement: Supplementary file 3 — Appendix 3. Acknowledgements. Text. (PDF 104 kb) [file 12913_2018_2915_MOESM3_ESM.pdf]

Acknowledgments
NHMRC Partnership Project APP1029492

Project Management Committee:

Roberto Forero* [1], Ken Hillman [1], Daniel Fatovich [2,3], Sally McCarthy [3,4], David Mountain [3,5], Peter Sprivulis [3,5], Antonio Celenza [3,5], Paul Tridgell [6], Mohammed Mohsin [7,8], Frank Daly [3,9], Elizabeth Rohwedder[10], Sam Green [10], Sarah Marmara [11], Gerard Fitzgerald [3,12], John Burke [3,13], Paul Middleton [3,14], Drew Richardson [3,15]

Associate Investigators:

Nick Gibson [16], Jeffrey Braithwaite [17], Peter Nugus [18]

Staff:

Hanh Ngo [5], Wing (Nicola) Man [1], Shizar Nahidi,[1] Sam Toloo, [12,] Josephine De Costa, [1] Fenglian Xu, [1] and Brydan Lenne [1]

1 *Simpson Centre for Health Services Research, **University of New South Wales**, Kensington,*

2 *Centre for Clinical Research in Emergency Medicine, Department of Emergency Medicine, **Royal Perth Hospital and University of Western Australia**, Perth, WA*

3 ***Australasian College for Emergency Medicine**, West Melbourne, VIC.*

4 ***Emergency Care Institute, NSW Agency for Clinical Innovation**, Chatswood, Sydney, NSW.*

5 *Emergency Medicine, **University of Western Australia**, Crawley, WA,*

6 ***Paul Tridgell Consulting**, Kenthurst, NSW,*

7 ***Psychiatry Research and Teaching Unit, SWSLHD, NSW Health** , Liverpool, NSW,*

8 *School of Public Health and Community Medicine, **UNSW**, Randwick, NSW,*

9 *South Metropolitan Health Service, **Department of Health Western Australia**, Mount Pleasant, WA,*

10 ***Health System Improvement Unit**, **Department of Health Western Australia**, East Perth, WA*

11 ***NSW Ministry of Health**, North Sydney, NSW.*

12 *School of Public Health and Social Work, **Queensland University of Technology**, Kelvin Grove, QLD,*

13 *Department of Emergency Medicine, **Royal Brisbane and Women's Hospital**, Herston, QLD,*

14 ***Australian Resuscitation Council of NSW**, NSW*

15 ***Australian National University Medical School and Canberra Hospital** ED, Canberra, ACT*

16 *Edith Cowan University, Perth, WA*

17 *Macquarie University, Sydney, NSW*

18 *McGill University, Montreal, Canada*
